# Structure and spectroscopic properties of *N*,*S*-coordinating 2-methyl­sulfanyl-*N*-[(1*H*-pyrrol-2-yl)methyl­idene]aniline methanol monosolvate

**DOI:** 10.1107/S205698901501590X

**Published:** 2015-09-12

**Authors:** D. Douglas Richards, M. Trisha C. Ang, Robert McDonald, Matthias Bierenstiel

**Affiliations:** a Department of Chemistry, 1250 Grand Lake Road, Cape Breton University, Sydney, Nova Scotia, B1P 6L2, Canada; b University of Alberta, X-ray Crystallography Laboratory, Department of Chemistry, Edmonton, Alberta, T6G 2G2, Canada

**Keywords:** crystal structure, imine, Schiff base, hydrogen bonding, *N*,*S*-ligand

## Abstract

The reaction of pyrrole-2-carboxaldehyde and 2-(methyl­sulfan­yl)aniline in refluxing methanol gave an olive-green residue in which yellow crystals of the title compound, C_12_H_12_N_2_S·CH_3_OH, were grown from slow evaporation of methanol at 263 K. In the crystal, hydrogen-bonding inter­actions link the aniline mol­ecule and a nearby methanol solvent mol­ecule. These units are linked by a pair of weak C—H⋯O_methanol_ interactions, forming inversion dimers consisting of two main molecules and two solvent molecules.

## Abstract 2

The title Schiff base, 2-methyl­sulfanyl-*N*-[(1*H*-pyrrol-2-yl)methyl­idene]aniline, crystallizes in the presence of a methanol mol­ecule and features three distinct hydrogen bonds to each heteroatom in the mol­ecule. The crystal lattice exhibits an array of methanol mol­ecules sandwiched between the title compound exhibiting weak supra­molecular inter­actions.

## Chemical context   

Compounds that contain N- and S-donor atoms have exhibited biomedical activities such as anti­bacterial properties. In addition, such *N,S*-compounds can be useful ligands to form transition metal complexes which we have been investigating for their use as biomimetic models for Cu enzyme models (Alberto *et al.*, 2013 ▸; Cross *et al.*, 2011 ▸). Recently, we have reported the synthesis and structure of xylylene-bridged *bis*-[*ortho*-amino­thio­phenols] for the design of binuclear transition metal complexes (Alberto *et al.*, 2013 ▸; Cross *et al.*, 2011 ▸). Copper complexes of these N_2_S_2_-ligands are studied as small biomimetic metal models for the analysis of non-blue/type-II copper enzymes such as peptidylglycine α-hy­droxy­lating monooxygenase (PHM), which is one of the two non-coupled copper ion domains of the bifunctional peptidylglycine α-amidating monooxygenase (PAM, EC 1.14.17.3) (Klinman, 2006 ▸; McIntyre *et al.*, 2009 ▸). Recently, we reported the X-ray structure of a trinuclear palladium(II) complex containing *N,S*-coordinating 2-(benzyl­sulfan­yl)anilinide and 1,3-benzo­thio­azole-2-thiol­ate ligands (Cross *et al.*, 2014 ▸). The 2-amino­thio­phenol group can be used as a synthetic building motif for the preparation of benzo­thia­zolines (Chou *et al.*, 2008 ▸), thio­ethers (Ham *et al.*, 2006 ▸; Schwindt *et al.*, 1976*a* ▸) and polyurethanes (Schwindt *et al.*, 1976*b* ▸), and has medical applications in anti­trypanosomal, anti­leishmanial and anti­malarial treatments (Parveen *et al.*, 2005 ▸).
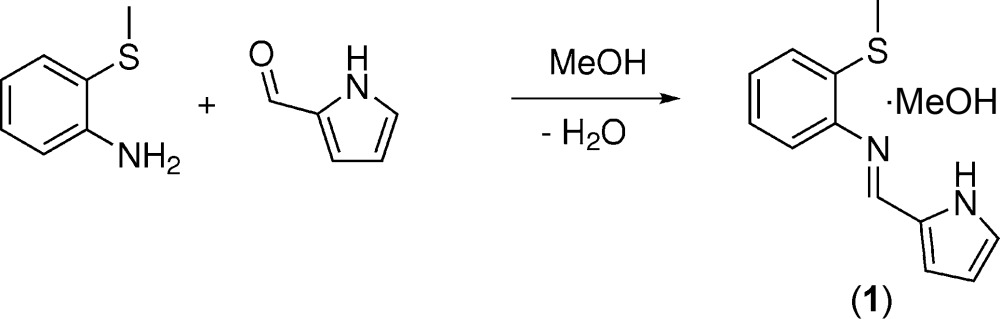


Herein, we report the X-ray structure of 2-methyl­sulfanyl-*N*-[(1*H*-pyrrol-2-yl)methyl­idene]aniline methanol monosolvate (**1**) which features an aryl methyl thio­ether group and an imino-2-pyrrole motif. The imine pendant prevents the reversible formation of the benzo­thia­zoline, a transformation that was evident in the structure we reported previously that featured a free amino group and was bonded to a palladium centre (Setifi *et al.*, 2014 ▸). Li and co-workers first described the synthesis of (**1**) in 16% isolated yield (He *et al.*, 2009 ▸). The ^1^H and ^13^C NMR data were reported and are congruent with our data (Basuli *et al.*, 1996 ▸). Compound (**1**) was complexed with CrCl_3_(thf)_3_ (He *et al.*, 2009 ▸) and with VCl_3_(thf)_3_ (Mu *et al.*, 2011 ▸) as ethyl­ene polymerization catalysts (He *et al.*, 2009 ▸). We now provide additional compound data such as HRMS, UV–vis and FT–IR.

## Structural commentary   

**Figure 1:**
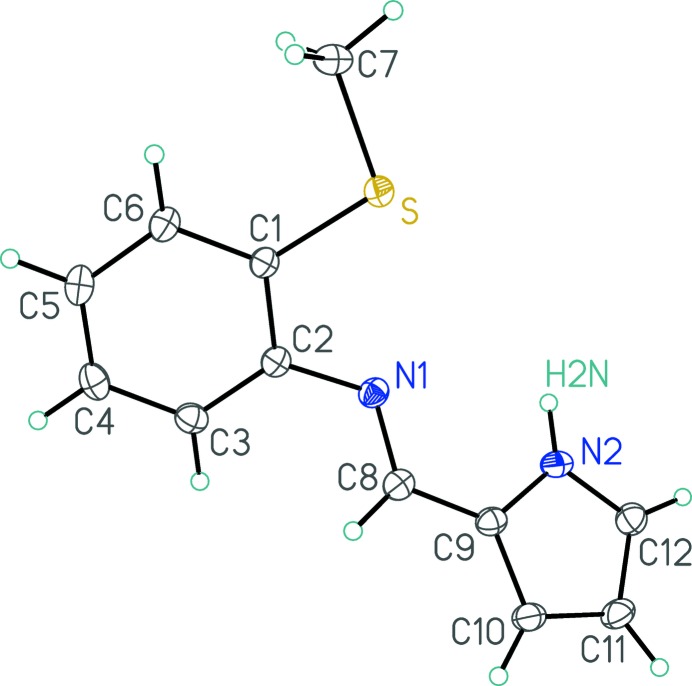
Perspective view of the 2-methyl­sulfanyl-*N*-[(1*H*-pyrrol-2-yl)methyl­idene]aniline mol­ecule showing the atom-labelling scheme. Non-hydrogen atoms are represented by Gaussian ellipsoids at the 30% probability level.

Fig. 1 ▸ shows the mol­ecular structure of the yellow title compound. The imino group is coplanar with the pyrrole group, and the dihedral angle between the plane of the combined (pyrrol-2-yl)imino moiety and that of the benzene ring carbons is 42.71 (5)°. The imino group N1–C8 bond distance [1.2829 (17) Å] is normal. The sulfur and imino nitro­gen atoms are very nearly coplanar with the benzene ring atoms [S is 0.0595 (18) Å and N1 is 0.0620 (19) Å out of plane], while the methyl carbon C7 is 0.310 (3) Å out of the benzene ring plane.

**Figure 2:**
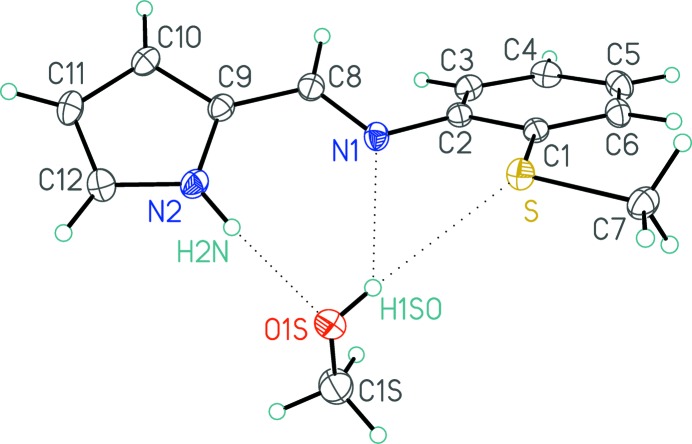
Illustration of hydrogen-bonded inter­actions (dotted lines) between the 2-methyl­sulfanyl-*N*-[(1*H*-pyrrol-2-yl)methyl­idene]aniline mol­ecule and a nearby solvent methanol mol­ecule.

The three heteroatoms of the main mol­ecule of (**1**) are each involved in hydrogen-bonding inter­actions with the adjacent co-crystallized solvent methanol mol­ecule (Fig. 2 ▸ and Table 1 ▸). The closest inter­action is between the protonated nitro­gen of the pyrrol-2-yl group and the methanol oxygen [N2⋯O1*S* = 2.9030 (16) Å; H2*N*⋯O1*S* = 2.025 (18) Å]. The methanol hydroxyl group shows somewhat weaker inter­actions with the imino nitro­gen [N1⋯H1*SO* = 2.49 (2) Å; N1⋯O1*S* = 3.1116 (16) Å] and the sulfur atom [S1⋯H1*SO* = 2.76 (3) Å; S⋯O1*S* = 3.5134 (12) Å].

## Supra­molecular features   

**Figure 3:**
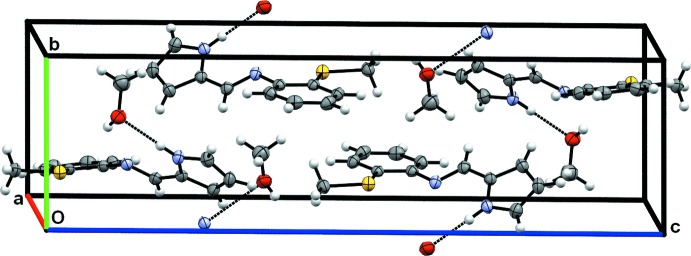
Packing view of (**1**) as viewed slightly offset from along the *a* axis.

As shown in Fig. 3 ▸, the methanol mol­ecules are sandwiched between the main mol­ecules of (**1**) in such a manner as to preclude π–π stacking inter­actions between aromatic rings of adjacent mol­ecules. The hydrogen-bonded methanol–main molecule units are linked by pairs of weakC—H⋯O_methanol_ inter­actions, forming inversion dimers consisting of two main molecules and two solvent molecules (Table 1 ▸). 

In summary, 2-methyl­sulfanyl-*N*-[(1*H*-pyrrol-2-yl)methyl­idene]aniline is a conjugated imine that exhibits three hydrogen-bonding inter­actions to methanol within the crystal packing which would make the compound effective for tridentate *N,N,S* metal chelation, particularly in the case where the *N-*hydrogen of the pyrrol-2-yl group is deproton­ated to form an anionic species.

## Thio­ether bonding in related structures   

This is the first crystallographic report of an NNS ligand system found in 2-methyl­sulfanyl-*N*-[(1*H*-pyrrol-2-yl)methyl­idene]aniline. The closest related structure to (**1**) is the reported mol­ecular structure of 3-(imino-*N*-2-methyl­sulfanylphen­yl)imidazo[1,5-*a*]pyridinium-1-thiol­ate (Patra *et al.*, 2011*a* ▸), where the imine-carbon atom is α to a nitro­gen heteroatom and crystallizes in space group *P* 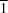. Related NNS-type ligands are published with their respective metal complexes.

A closely related compound that features a pyridyl group instead of a pyrrole has been extensively reported in metal complexes and whether the thio­ether bonds to the metal centre varies, which sheds perspective on the binding nature of compound (**1**). For example, the thio­ether of the pyridyl ligand does not initially bind to the metal centre of a manganese carbonyl complex unless in the presence of oxygen (Lumsden *et al.*, 2014 ▸). When reacted with a rhenium carbonyl complex (Jana *et al.*, 2013 ▸), the thio­ether does not participate in bonding, and in contrast, the thio­ether binds to iron in its respective carbonyl complex (Mu­thiah *et al.*, 2015 ▸).

This variance in thio­ether bonding is also found when reacting the pyridyl ligand with various copper complexes (Addison *et al.*, 1984 ▸; Schnödt *et al.*, 2011 ▸; Patra *et al.*, 2011*b* ▸; Chatterjee *et al.*, 2012 ▸; Balamurugan *et al.*, 2006 ▸) where copper is our target metal centre for (**1**) and for our other NNS ligands. Addison and co-workers have reported a systematic study on the properties of various copper–thio­ether inter­actions (Addison *et al.*, 1984 ▸). In the study, they considered the presence of a nitro­gen donor in an equatorial plane to the thio­ether, strong donor solvents, and the redox chemistry of the resultant metal complexes, which would affect the displacement of the thio­ether group.

The methanol mol­ecule present in the X-ray structure of (**1**) does illustrate the three heteroatoms that could bond to a metal centre, though perspective can be gained from the metal complexes formed with the pyridyl ligand relative. The reported mol­ecular structures with the pyridyl metal complexes all feature distorted octa­hedral geometry. In the case of (**1**), the thio­ether group sits above the neighbouring benzene ring, which would contribute to the formation of a distorted octa­hedral complex and remove the direct equatorial inter­action of the sulfur to the donating nitro­gen of the imine group. In addition, the pyrrole substituent is relatively less basic than pyridine, hence deprotonation of the pyrrole ligand must occur to elicit coordination of a metal centre.

## Synthesis and crystallization   

All chemicals were purchased from commercial sources (Fisher Scientific and Sigma–Aldrich) and used without further purification. A colorless solution containing 0.683 g (7.18 mmol) of 2-pyrrole­carboxaldehyde dissolved in 15 mL of MeOH was added drop-wise to a light-green solution containing 1.00 g (7.18 mmol) of 2-(methyl­sulfan­yl)aniline in 5 mL of MeOH with stirring. After refluxing the light-green solution overnight, the solution changed color to olive green. The solution was cooled to ambient temperature, and the solvent was removed under reduced pressure. The olive-green residue was dissolved in 10 mL of MeOH, and was placed in the freezer at 263 K with a needle-punctured rubber septum. Crystals formed from the solution, and, after several days, were collected by vacuum filtration and washed with cold hexa­nes. 0.780 g (50%) of yellow crystals were isolated.

## Spectroscopic investigations   

NMR spectra were recorded on a Bruker Avance II 400 MHz spectrometer operating at 400.17 MHz for ^1^H and 100.6 MHz for ^13^C, and were referenced to tetra­methyl­silane (δ = 0 p.p.m.). High-resolution MS data were obtained using a Waters XevoG2 QToF instrument in positive electrospray ionization mode. Theoretical *m*/*z* values are reported for an abundance greater than 10% of base signal. UV-Vis spectra were recorded in quartz cuvettes on a Varian Cary 100 Bio UV–Vis spectrometer. FT–IR spectra were recorded on a Thermo Nicolet 6700 FT–IR Spectrometer as KBr pellet (approximately 1.5 mg compound in 300 mg anhydrous KBr) in the 4,000 cm^−1^ to 400 cm^−1^ range with 2 cm^−1^ resolution.

Spectroscopic measurements confirmed the structure of (**1**). High-resolution mass spectrometry gave an [*M*]^+^ ion of 217.0833 *m*/*z*, close to the calculated mass of 217.0755 *m*/*z and* the IR spectrum of (**1**) exhibited an imine stretch of 1611 cm^−1^ that is characteristic for aniline-based imines. Absorbances located at 290 nm and 300 nm in the UV spectrum are characteristic of the π–π* transition of pyrrole and C=N bonds, respectively. The π–π* transition of benzene is also present with an absorbance around 350 nm.

^1^H NMR (400 MHz, CDCl_3_) δ = 9.63 (*bs*, 1H), 8.22 (*s*, 1H), 7.22–7.14 (*m*, 3H), 7.03 (*bs*, 1H), 6.99 (*d*, *J* = 7.6 Hz, 1H), 6.71 (*dd*, *J* = 1.2, 3.6 Hz, 1H), 6.34 (*t*, *J* = 2.8 Hz, 1H), 2.47 (*s*, 3H) p.p.m.^13^C{^1^H} NMR (100 MHz, CDCl_3_) δ = 149.1, 148.9, 133.9, 131.0, 125.9, 125.2, 124.3, 123.0, 117.5, 116.4, 110.6, 14.7 p.p.m. FT–IR (KBr) 3264, 3154, 2934, 3127, 3109. 3079, 3060, 2980, 2968, 2912, 2890, 2854, 1611, 1573, 1568, 1550, 1470, 1450, 1439, 1418, 1333, 1308, 1267, 1246, 1205, 1133, 1094, 1070, 1038, 975, 970, 956, 927, 882, 864, 844, 829, 781, 746, 725, 678, 603, 586 cm^−1^. HRMS (ESI–TOF) *m*/*z*: [*M*]^+^ Calculated for C_12_H_12_N_2_S 217.0755; found 217.0833. *λ*_max_/nm (DMF, 0.022 mg mL^−1^) 303 (*λ*/dm^3^ mol^−1^cm^−1^ 21700), 270 (19100), 208 (*sh*).

## Refinement details   

Crystal data, data collection and structure refinement details are summarized in Table 2 ▸. Hydrogen atoms attached to carbons were assigned positions based on the *sp* ^2^ or *sp* ^3^ hybridization geometries of their attached atoms. Hydrogens attached to *sp* ^2^-hybridized carbons were given isotropic displacement parameters *U*_iso_(H) = 1.2*U*_iso_(C) for their attached atoms, while methyl-group hydrogens were given isotropic displacement parameters *U*_iso_(H) = 1.5*U*_eq_(C) for their attached carbons. The coordin­ates and displacement parameters for the hydrogens attached to N2 and O1*S* were allowed to refine freely.

## Supplementary Material

Crystal structure: contains datablock(s) I, New_Global_Publ_Block. DOI: 10.1107/S205698901501590X/bg2566sup1.cif

Structure factors: contains datablock(s) I. DOI: 10.1107/S205698901501590X/bg2566Isup2.hkl

Supporting information file. DOI: 10.1107/S205698901501590X/bg2566Isup3.cml

CCDC reference: 1417853

Additional supporting information:  crystallographic information; 3D view; checkCIF report

## Figures and Tables

**Table 1:** Hydrogen-bond geometry (, )

*D*H*A*	*D*H	H*A*	*D* *A*	*D*H*A*
N2H2*N*O1*S*	0.884(18)	2.025(18)	2.9030(16)	172.0(16)
O1*S*H1*SO*S	0.83(3)	2.76(3)	3.5134(12)	152(2)
O1*S*H1*SO*N1	0.83(3)	2.49(2)	3.1116(16)	132(2)
C7H7*B*O1*S* ^i^	0.98	2.55	3.5181(18)	168

Symmetry code: (i) 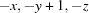.

**Table 2:** Experimental details

Crystal data	
Chemical formula	C_12_H_12_N_2_SCH_4_O
*M*_r_	248.34
Crystal system, space group	Monoclinic, *P*2_1_/*c*
Temperature (K)	193
*a*, *b*, *c* ()	7.5959(4), 7.0062(4), 24.4986(14)
()	98.4543(7)
*V* (^3^)	1289.61(12)
*Z*	4
Radiation type	Mo *K*
(mm^1^)	0.24
Crystal size (mm)	0.24 0.20 0.15

Data collection	
Diffractometer	Bruker APEXII CCD
Absorption correction	Integration (*SADABS*; Bruker, 2013 ▸)
*T*_min_, *T*_max_	0.935, 1.000
No. of measured, independent and observed [*I* > 2(*I*)] reflections	10802, 3055, 2579
*R*_int_	0.025
(sin /)_max_ (^1^)	0.663

Refinement	
*R*[*F* ^2^ > 2(*F* ^2^)], *wR*(*F* ^2^), *S*	0.032, 0.090, 1.04
No. of reflections	3055
No. of parameters	165
H-atom treatment	H atoms treated by a mixture of independent and constrained refinement
_max_, _min_ (e ^3^)	0.28, 0.20

Computer programs: *APEX2* and *SAINT* (Bruker, 2013 ▸), *SHELXD* (Schneider Sheldrick, 2002 ▸), *SHELXL2014* (Sheldrick, 2015 ▸), *SHELXTL* (Sheldrick, 2008 ▸) and *Mercury* (Macrae *et al.*, 2006 ▸).

## supplementary crystallographic information

### Crystal data

**Table:**

C_12_H_12_N_2_S·CH_4_O	*F*(000) = 528
*M**_r_* = 248.34	*D*_x_ = 1.279 Mg m^−^^3^
Monoclinic, *P*2_1_/*c*	Mo *K*α radiation, λ = 0.71073 Å
*a* = 7.5959 (4) Å	Cell parameters from 5994 reflections
*b* = 7.0062 (4) Å	θ = 2.7–27.9°
*c* = 24.4986 (14) Å	µ = 0.24 mm^−^^1^
β = 98.4543 (7)°	*T* = 193 K
*V* = 1289.61 (12) Å^3^	Fragment, colourless
*Z* = 4	0.24 × 0.20 × 0.15 mm

### Data collection

**Table:**

Bruker APEXII CCD diffractometer	2579 reflections with *I* > 2σ(*I*)
ω scans	*R*_int_ = 0.025
Absorption correction: integration (*SADABS*; Bruker, 2013)	θ_max_ = 28.1°, θ_min_ = 1.7°
*T*_min_ = 0.935, *T*_max_ = 1.000	*h* = −9→9
10802 measured reflections	*k* = −9→9
3055 independent reflections	*l* = −31→32

### Refinement

**Table:**

Refinement on *F*^2^	Hydrogen site location: mixed
Least-squares matrix: full	H atoms treated by a mixture of independent and constrained refinement
*R*[*F*^2^ > 2σ(*F*^2^)] = 0.032	*w* = 1/[σ^2^(*F*_o_^2^) + (0.0414*P*)^2^ + 0.4071*P*] where *P* = (*F*_o_^2^ + 2*F*_c_^2^)/3
*wR*(*F*^2^) = 0.090	(Δ/σ)_max_ < 0.001
*S* = 1.04	Δρ_max_ = 0.28 e Å^−^^3^
3055 reflections	Δρ_min_ = −0.20 e Å^−^^3^
165 parameters	Extinction correction: *SHELXL2014* (Sheldrick, 2015), Fc^*^=kFc[1+0.001xFc^2^λ^3^/sin(2θ)]^-1/4^
0 restraints	Extinction coefficient: 0.0044 (11)

### Special details

**Table:**

Geometry. All esds (except the esd in the dihedral angle between two l.s. planes) are estimated using the full covariance matrix. The cell esds are taken into account individually in the estimation of esds in distances, angles and torsion angles; correlations between esds in cell parameters are only used when they are defined by crystal symmetry. An approximate (isotropic) treatment of cell esds is used for estimating esds involving l.s. planes.

### Fractional atomic coordinates and isotropic or equivalent isotropic displacement parameters (Å^2^)

**Table:**

	*x*	*y*	*z*	*U*_iso_*/*U*_eq_	
S	0.27868 (4)	0.27649 (5)	0.03023 (2)	0.02884 (11)	
N1	0.43422 (14)	0.29520 (16)	0.14334 (4)	0.0274 (2)	
N2	0.19082 (15)	0.38459 (17)	0.21958 (5)	0.0308 (3)	
H2N	0.169 (2)	0.457 (3)	0.1897 (7)	0.049 (5)*	
C1	0.50824 (16)	0.24857 (17)	0.05218 (5)	0.0242 (3)	
C2	0.56495 (17)	0.25724 (17)	0.10935 (5)	0.0255 (3)	
C3	0.74554 (18)	0.24302 (19)	0.12952 (6)	0.0309 (3)	
H3	0.7846	0.2513	0.1681	0.037*	
C4	0.86910 (17)	0.2168 (2)	0.09371 (6)	0.0338 (3)	
H4	0.9922	0.2080	0.1078	0.041*	
C5	0.81268 (18)	0.2034 (2)	0.03767 (6)	0.0340 (3)	
H5	0.8972	0.1837	0.0132	0.041*	
C6	0.63324 (17)	0.21859 (19)	0.01680 (6)	0.0296 (3)	
H6	0.5955	0.2085	−0.0218	0.036*	
C7	0.2639 (2)	0.2898 (2)	−0.04355 (6)	0.0345 (3)	
H7A	0.3419	0.3918	−0.0533	0.052*	
H7B	0.1408	0.3171	−0.0598	0.052*	
H7C	0.3011	0.1678	−0.0578	0.052*	
C8	0.44496 (18)	0.21148 (19)	0.19028 (5)	0.0289 (3)	
H8	0.5356	0.1188	0.1998	0.035*	
C9	0.32524 (18)	0.25263 (19)	0.22884 (5)	0.0280 (3)	
C10	0.31947 (19)	0.1746 (2)	0.28029 (5)	0.0330 (3)	
H10	0.3969	0.0790	0.2977	0.040*	
C11	0.1793 (2)	0.2613 (2)	0.30227 (6)	0.0362 (3)	
H11	0.1440	0.2356	0.3372	0.043*	
C12	0.10244 (19)	0.3905 (2)	0.26377 (5)	0.0348 (3)	
H12	0.0039	0.4705	0.2675	0.042*	
O1S	0.15530 (14)	0.62192 (16)	0.12144 (4)	0.0388 (3)	
H1SO	0.221 (3)	0.551 (4)	0.1064 (10)	0.090 (8)*	
C1S	0.2439 (2)	0.7991 (2)	0.12954 (7)	0.0493 (4)	
H1SA	0.2039	0.8659	0.1607	0.074*	
H1SB	0.2167	0.8768	0.0961	0.074*	
H1SC	0.3726	0.7775	0.1375	0.074*	

### Atomic displacement parameters (Å^2^)

**Table:**

	*U*^11^	*U*^22^	*U*^33^	*U*^12^	*U*^13^	*U*^23^
S	0.02473 (17)	0.0349 (2)	0.02682 (18)	−0.00058 (12)	0.00369 (12)	−0.00038 (13)
N1	0.0292 (5)	0.0298 (6)	0.0235 (5)	−0.0037 (4)	0.0048 (4)	−0.0030 (4)
N2	0.0348 (6)	0.0332 (6)	0.0238 (5)	0.0001 (5)	0.0020 (4)	0.0037 (5)
C1	0.0252 (6)	0.0213 (6)	0.0265 (6)	−0.0033 (4)	0.0054 (5)	−0.0004 (5)
C2	0.0274 (6)	0.0223 (6)	0.0274 (6)	−0.0025 (5)	0.0055 (5)	−0.0006 (5)
C3	0.0304 (6)	0.0302 (7)	0.0310 (7)	−0.0018 (5)	0.0007 (5)	0.0017 (5)
C4	0.0236 (6)	0.0322 (7)	0.0452 (8)	−0.0007 (5)	0.0035 (5)	−0.0003 (6)
C5	0.0292 (6)	0.0333 (8)	0.0421 (8)	−0.0020 (5)	0.0135 (6)	−0.0056 (6)
C6	0.0304 (6)	0.0305 (7)	0.0294 (7)	−0.0040 (5)	0.0089 (5)	−0.0042 (5)
C7	0.0400 (7)	0.0345 (8)	0.0273 (7)	−0.0033 (6)	−0.0006 (5)	0.0031 (5)
C8	0.0309 (6)	0.0283 (7)	0.0274 (6)	−0.0039 (5)	0.0043 (5)	−0.0017 (5)
C9	0.0313 (6)	0.0276 (7)	0.0246 (6)	−0.0035 (5)	0.0028 (5)	0.0002 (5)
C10	0.0367 (7)	0.0346 (7)	0.0271 (7)	−0.0008 (6)	0.0029 (5)	0.0058 (5)
C11	0.0413 (8)	0.0446 (8)	0.0234 (6)	−0.0034 (6)	0.0072 (5)	0.0013 (6)
C12	0.0346 (7)	0.0392 (8)	0.0312 (7)	0.0007 (6)	0.0068 (5)	−0.0027 (6)
O1S	0.0424 (6)	0.0343 (6)	0.0414 (6)	0.0066 (5)	0.0124 (5)	0.0013 (5)
C1S	0.0525 (9)	0.0449 (10)	0.0524 (10)	−0.0038 (8)	0.0144 (8)	−0.0030 (8)

### Geometric parameters (Å, º)

**Table:**

S—C1	1.7586 (13)	C7—H7A	0.9800
S—C7	1.7968 (14)	C7—H7B	0.9800
N1—C8	1.2829 (17)	C7—H7C	0.9800
N1—C2	1.4118 (16)	C8—C9	1.4334 (18)
N2—C12	1.3557 (17)	C8—H8	0.9500
N2—C9	1.3714 (17)	C9—C10	1.3806 (18)
N2—H2N	0.884 (18)	C10—C11	1.401 (2)
C1—C6	1.3924 (17)	C10—H10	0.9500
C1—C2	1.4049 (18)	C11—C12	1.374 (2)
C2—C3	1.3915 (18)	C11—H11	0.9500
C3—C4	1.388 (2)	C12—H12	0.9500
C3—H3	0.9500	O1S—C1S	1.412 (2)
C4—C5	1.380 (2)	O1S—H1SO	0.83 (3)
C4—H4	0.9500	C1S—H1SA	0.9800
C5—C6	1.3878 (19)	C1S—H1SB	0.9800
C5—H5	0.9500	C1S—H1SC	0.9800
C6—H6	0.9500		
			
C1—S—C7	103.05 (7)	S—C7—H7C	109.5
C8—N1—C2	119.01 (12)	H7A—C7—H7C	109.5
C12—N2—C9	109.42 (11)	H7B—C7—H7C	109.5
C12—N2—H2N	126.0 (11)	N1—C8—C9	122.40 (13)
C9—N2—H2N	124.6 (11)	N1—C8—H8	118.8
C6—C1—C2	119.34 (12)	C9—C8—H8	118.8
C6—C1—S	124.24 (10)	N2—C9—C10	107.17 (12)
C2—C1—S	116.42 (9)	N2—C9—C8	123.75 (12)
C3—C2—C1	119.51 (12)	C10—C9—C8	129.07 (13)
C3—C2—N1	123.11 (12)	C9—C10—C11	107.89 (13)
C1—C2—N1	117.19 (11)	C9—C10—H10	126.1
C4—C3—C2	120.54 (13)	C11—C10—H10	126.1
C4—C3—H3	119.7	C12—C11—C10	106.98 (12)
C2—C3—H3	119.7	C12—C11—H11	126.5
C5—C4—C3	119.86 (13)	C10—C11—H11	126.5
C5—C4—H4	120.1	N2—C12—C11	108.53 (13)
C3—C4—H4	120.1	N2—C12—H12	125.7
C4—C5—C6	120.39 (13)	C11—C12—H12	125.7
C4—C5—H5	119.8	C1S—O1S—H1SO	106.4 (18)
C6—C5—H5	119.8	O1S—C1S—H1SA	109.5
C5—C6—C1	120.32 (13)	O1S—C1S—H1SB	109.5
C5—C6—H6	119.8	H1SA—C1S—H1SB	109.5
C1—C6—H6	119.8	O1S—C1S—H1SC	109.5
S—C7—H7A	109.5	H1SA—C1S—H1SC	109.5
S—C7—H7B	109.5	H1SB—C1S—H1SC	109.5
H7A—C7—H7B	109.5		
			
C7—S—C1—C6	−7.76 (13)	C2—C1—C6—C5	−2.00 (19)
C7—S—C1—C2	172.43 (10)	S—C1—C6—C5	178.18 (10)
C6—C1—C2—C3	2.43 (18)	C2—N1—C8—C9	175.38 (11)
S—C1—C2—C3	−177.75 (10)	C12—N2—C9—C10	0.08 (15)
C6—C1—C2—N1	177.62 (11)	C12—N2—C9—C8	−179.73 (13)
S—C1—C2—N1	−2.55 (15)	N1—C8—C9—N2	−0.6 (2)
C8—N1—C2—C3	−41.88 (18)	N1—C8—C9—C10	179.65 (14)
C8—N1—C2—C1	143.11 (12)	N2—C9—C10—C11	−0.04 (16)
C1—C2—C3—C4	−1.2 (2)	C8—C9—C10—C11	179.75 (13)
N1—C2—C3—C4	−176.13 (12)	C9—C10—C11—C12	−0.01 (16)
C2—C3—C4—C5	−0.4 (2)	C9—N2—C12—C11	−0.08 (16)
C3—C4—C5—C6	0.9 (2)	C10—C11—C12—N2	0.06 (17)
C4—C5—C6—C1	0.4 (2)		

### Hydrogen-bond geometry (Å, º)

**Table:**

*D*—H···*A*	*D*—H	H···*A*	*D*···*A*	*D*—H···*A*
N2—H2*N*···O1*S*	0.884 (18)	2.025 (18)	2.9030 (16)	172.0 (16)
O1*S*—H1*SO*···S	0.83 (3)	2.76 (3)	3.5134 (12)	152 (2)
O1*S*—H1*SO*···N1	0.83 (3)	2.49 (2)	3.1116 (16)	132 (2)
C7—H7*B*···O1*S*^i^	0.98	2.55	3.5181 (18)	168

Symmetry code: (i) −*x*, −*y*+1, −*z*.
